# Polymerization and editing modes of a high-fidelity DNA polymerase are linked by a well-defined path

**DOI:** 10.1038/s41467-020-19165-2

**Published:** 2020-10-23

**Authors:** Thomas Dodd, Margherita Botto, Fabian Paul, Rafael Fernandez-Leiro, Meindert H. Lamers, Ivaylo Ivanov

**Affiliations:** 1 Department of Chemistry, Georgia State University, Atlanta, GA USA; 2 Center for Diagnostics and Therapeutics, Georgia State University, Atlanta, GA USA; 3 Department of Cell and Chemical Biology, Leiden University Medical Center, Leiden, The Netherlands; 4 Department of Biochemistry & Molecular Biology, University of Chicago, Chicago, IL USA; 5 Spanish National Cancer Research Centre (CNIO), Madrid, Spain

**Keywords:** Biochemistry, Computational biophysics, DNA synthesis

## Abstract

Proofreading by replicative DNA polymerases is a fundamental mechanism ensuring DNA replication fidelity. In proofreading, mis-incorporated nucleotides are excised through the 3′-5′ exonuclease activity of the DNA polymerase holoenzyme. The exonuclease site is distal from the polymerization site, imposing stringent structural and kinetic requirements for efficient primer strand transfer. Yet, the molecular mechanism of this transfer is not known. Here we employ molecular simulations using recent cryo-EM structures and biochemical analyses to delineate an optimal free energy path connecting the polymerization and exonuclease states of *E. coli* replicative DNA polymerase Pol III. We identify structures for all intermediates, in which the transitioning primer strand is stabilized by conserved Pol III residues along the fingers, thumb and exonuclease domains. We demonstrate switching kinetics on a tens of milliseconds timescale and unveil a complete pol-to-exo switching mechanism, validated by targeted mutational experiments.

## Abstract (web summary)

In high fidelity DNA polymerases the exonuclease site is distal from the polymerization site and it is unknown how the primer strand travels between the two sites when mis-incorporated nucleotides must be removed. Here, the authors perform MD simulations and identify an optimal path for DNA primer strand translocation in the *E. coli* replicative DNA polymerase III and characterise the kinetics and dynamics of the Pol III pol-to-exo mode transition, which is validated with mutagenesis experiments.

## Introduction

Replicative DNA polymerases synthesize new DNA with extraordinary fidelity^1,2^. Incorrect nucleotide insertion into the growing primer strand occurs at a rate not exceeding one per 10^6^ synthesized bases. Three distinct features of the DNA polymerase holoenzyme are responsible for this remarkable precision^3–6^. First, polymerases’ active sites have evolved to select for the nucleotide with correct Watson-Crick base pairing to the template strand. Second, after mismatch incorporation, the growing end of the primer terminus becomes misplaced, preventing further DNA extension. Third, the mismatch presence induces DNA fraying at the primer-template junction^7^, promoting release of the primer end from the polymerase active site. Together, this outcome alters the equilibrium between DNA synthesis (polymerization) and excision by the 3′–5′ exonuclease subunit (editing or exonuclease activity).

Removal of mis-incorporated nucleotides is essential for accurate genome duplication. Yet, the molecular mechanism of transferring the primer end from the polymerase to the exonuclease active sites remains elusive. In a recent breakthrough, cryo-EM captured the bacterial DNA polymerase III (Pol III) core in both the polymerase and exonuclease functional states^7,8^, shedding light on the conformational changes that must accompany pol-to-exo mode conformational switching. While informative, the new structures visualize only the end states of the switching transition and, thus, do not explain how the primer end traverses the ~60-Å distance separating the two active sites.

**Fig. 1:**
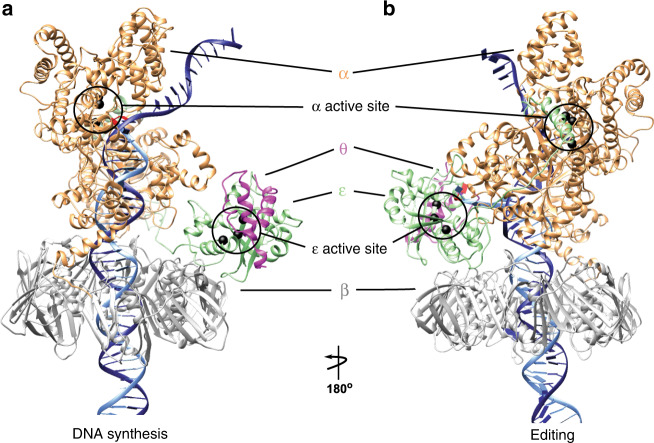
Structural models of DNA polymerase III in polymerization and editing mode. The Pol III core (α, ε, and θ) bound to the DNA sliding clamp β in (**a**) polymerization mode and (**b**) editing mode. Pol III subunits are colored and labeled as follows: α in orange, ε in green, θ in magenta, and β in gray. The primer and template DNA strands are shown in light and dark gray respectively. The polymerase active site (in the α subunit) and exonuclease active site (in the ε subunit) are highlighted with circles.

To understand the mechanism of this process vital for genome stability, we focus on the core of the *Escherichia coli* Pol III holoenzyme, composed of the α, ε, and θ subunits (Fig. 1). Similar to other C-family polymerases, the α subunit^9^ holds the polymerization site and has a characteristic shape resembling a right hand with fingers, thumb, and palm domains^10–12^. The α subunit also has a Polymerase and Histidinol Phosphatase (PHP) domain. Known to function as the exonuclease in most bacteria, the PHP has been inactivated in proteobacteria such as *E. coli*^13,14^. Instead, the ε subunit serves as the 3′–5′ exonuclease and is directly attached to α by the thumb and PHP domains^15,16^. The θ subunit has no enzymatic function but binds and stabilizes ε^17–19^. The Pol III core (α, ε, and θ) binds to the DNA sliding clamp β^20,21^, essential for processive DNA synthesis. DNA synthesis by Pol III core – β complex is fast (600–1000 nucleotides per second), processive (>100,000 nucleotides per binding event) and at the same time highly precise (error rate ~1 per million)^16,22–24^.

Modern computational science offers powerful tools to expose the microscopic dynamics underlying complex biomolecular transitions, provided that structures for the initial and final states are known. Specifically, in this study we relied on chain-of-replicas path optimization^25–28^ to compute a minimum free energy path connecting the polymerization and proofreading states of the Pol III holoenzyme, in which the DNA construct had a G:T mismatch at the primer end. Applying path optimization methods to large macromolecular complexes was, until recently, computationally prohibitively expensive. Advances in GPU technology and massively parallel computing platforms made it possible to use molecular dynamics (MD) to sample the conformational ensemble along the precomputed path (>6 μs of combined unbiased and biased sampling). We then employed the transition-based reweighting analysis method (TRAM) to construct a multi-ensemble Markov model (MEMM)^29,30^ from the MD trajectory data. The MEMM yields a complete kinetic model for the pol-to-exo mode conformational switching, including transition rates for all on-path intermediates. After partitioning the conformational ensemble into distinct kinetic macrostates, we applied dynamic network analysis to each macrostate. Key residues (critical nodes) along the path of the transitioning primer were determined, extending from the α subunit palm and thumb domain to the ε subunit. To validate the computational models, knowledge of the critical nodes was combined with data from conservation analysis to design mutations that disrupt the ordered transfer of the primer end to the exonuclease site and, thus, affect the balance between DNA synthesis and editing. Collectively, our results unravel the molecular origins of Pol III holoenzyme efficiency and fidelity.

## Results

### Pol III holoenzyme transitions from pol to exo mode along a well-defined path

To model the Pol III holoenzyme conformational transition from polymerization to editing, we started with the end point conformations captured by cryo-EM^7,8^. We built models for the two end states (denoted pol and exo, respectively), comprised of Pol III core, the β-clamp and primer-template DNA with a G-T mismatch at the primer end. We then used molecular dynamics flexible fitting (MDFF) with a weak scaling factor (*ξ* = 0.1) to extensively equilibrate the models, while ensuring conformance to the respective EM densities. A short targeted MD run was used to connect the equilibrated end states. From the targeted MD trajectory we selected 32 evenly spaced snapshots (replicas) that served to initiate our path optimization protocol, employing the partial nudged elastic band method (PNEB)^25,26^. In PNEB, the minimum energy path connecting protein functional states is represented by a series of replicas of the simulation system. PNEB uses forces from MD to optimize the protein conformations in all replicas to minimize the energy gradient perpendicular to the path. Forces applied parallel to the path keep the conformations in neighboring replicas distinct, while allowing the path to sample favorable regions of the free energy landscape. In this instance, we ran PNEB until convergence with 32 replicas representing the path, accumulating 18 ns of sampling per replica.

**Fig. 2:**
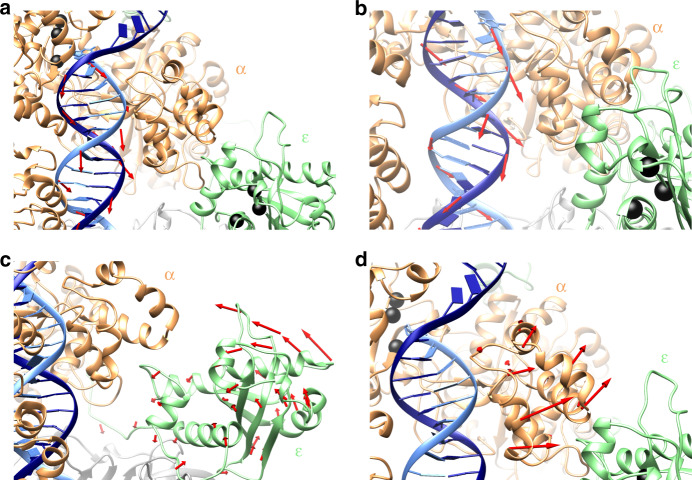
Concerted motions of Pol III holoenzyme guide the primer along the path toward the exonuclease state. **a** Initial backtracking motion of the DNA duplex away from polymerase active site observed in the MEP. **b** Subsequent rotational motion of the DNA and additional backtracking facilitates sequential unpairing at the primer/template junction. **c** Tilting motion of the ε subunit toward the α subunit shortens the distance between the polymerase and exonuclease active sites. **d** Outward shift of the thumb domain with respect to the PHP domain creates an opening to accommodate repositioning of the ε subunit. Red arrows indicate direction of motions observed in the minimum free energy path (MEP). Shifts in atomic positions for consecutive replicas of the MEP during different stages of the pol-to-exo transition were computed as vectors and mapped onto the structural elements of the Pol III holoenzyme. The α subunit is shown in orange; the ε subunit is shown in light green; the primer and template DNA strands are shown in light and dark blue, respectively; residues in the pol and exo active sites are shown as black spheres. The θ subunit has been omitted for clarity.

Our computed MEP (Supplementary Movie 1) delineates the sequence of molecular events and precise conformational shifts that transition the Pol III core from a pol to exo state. The process begins by fraying of the mismatched G-T pair at the primer terminus. To reach the exonuclease active site, three nucleotides must unpair at the primer-template junction and extend toward the ε subunit. Indeed, apart from G-T mismatch fraying, we observe two additional sequential unpairing events (Supplementary Fig. 1). However, DNA fraying and unpairing is not sufficient to accomplish this transition. In polymerization mode, a 3-nucleotide ssDNA overhang, even if fully extended, would not be able to span the ~70-Å distance to the exonuclease site (Supplementary Fig. 2). Instead, we observe 5.3 Å backtracking and 32.9° rotation of the DNA duplex that occupies the central cavity formed by the ring-shaped β-clamp and the Pol III core (Fig. 2a, b). Importantly, the Pol III core takes advantage of the spiral motion of dsDNA inside the cavity – a motion which is also essential for successful primer extension during replication. The Pol III core accommodates this motion by presenting positively charged residues along the entire length of the bound dsDNA (Supplementary Fig. 3)^8^. These transient contacts track along the DNA backbone and facilitate the forward rotational movement of Pol III during DNA synthesis. Upon encountering a mismatch, a continuation of the spiral motion, but without addition of nucleotides brings the fraying primer terminus in proximity to the exonuclease site. The third element necessary for pol-to-exo mode switching is the conformational change of the Pol III core itself. Specifically, in the minimum energy path we observe an ~12° tilting movement of the ε subunit toward the α subunit, which shortens the distance to the exo site by ~10 Å in the pol-to-exo mode transition (Fig. 2c). Furthermore, the thumb domain moves outwards to make space for the passing primer (Fig. 2d). While the thumb domain’s role as a steric wedge to separate the DNA strands is unique to the C-family of DNA polymerases, its functional significance has been highlighted in both A and B-family DNA polymerases^7,31,32^. Here, our computational modeling sheds light on a new role for the thumb domain, which is to create an opening to accommodate the shift of the ε subunit.

### Stable intermediates and a complete kinetic model for the pol-to-exo mode transition

PNEB optimization produces a time ordered series of structures, representing the Pol III pol-to-exo mode transition in its entirety. Next, we used these structures as seeds to initiate free molecular dynamics simulations and extensively sample the conformational ensemble along the optimal path. Since unbiased MD yields an ensemble obeying Boltzmann statistics, it becomes possible to analyze this ensemble and identify metastable states, corresponding to stable intermediates along the path. Moreover, the MD trajectories hold information on all observed state-to-state transitions, which allows us to construct kinetic models linking the on-path metastable states.

**Fig. 3:**
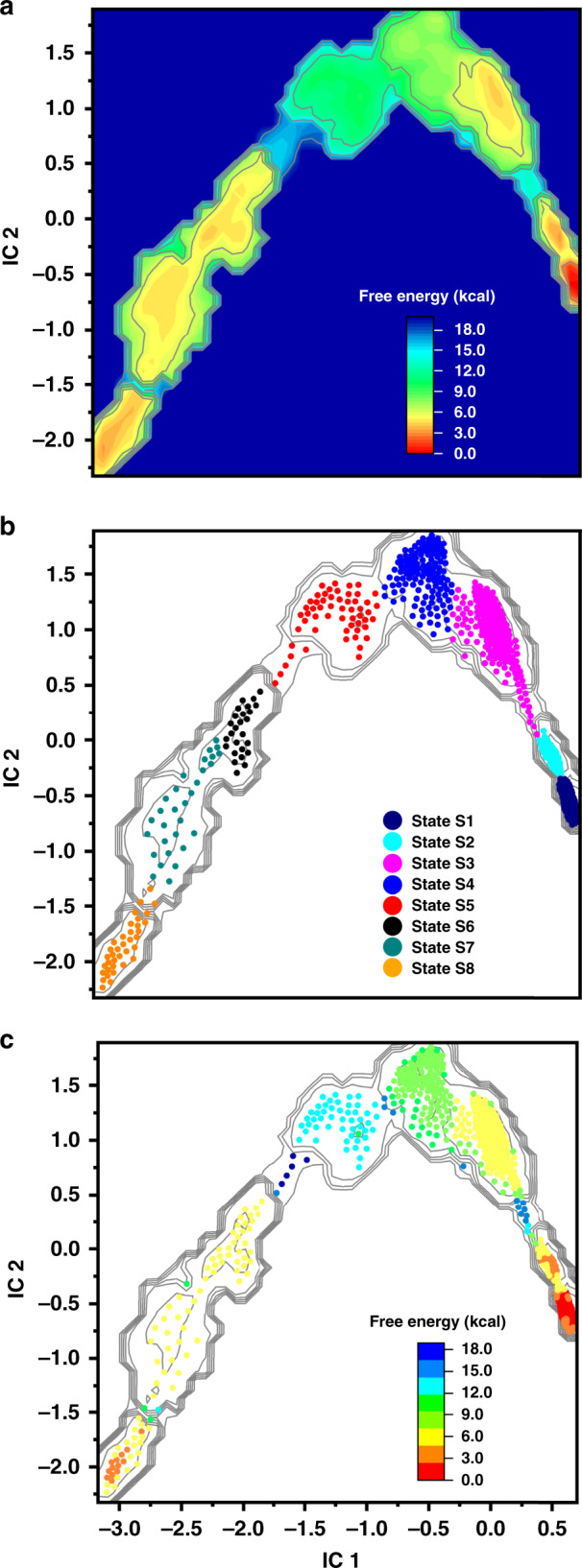
Analysis of the Pol III conformational ensemble reveals distinct kinetic intermediates in the pol-to-exo transition. **a** Effective free energy profile projected onto the first two independent components (ICs) from TICA analysis. Inset denotes ΔG scale in kcal/mol and is set relative to the polymerization state. **b** Multi-ensemble Markov model (MEMM) constructed by combining the biased and unbiased simulation ensembles. Microstates (dots) are colored by the macrostate (intermediate) they belong to. Macrostate identities were computed with the PCCA+ algorithm. Color scheme for the macrostates is shown in the inset. **c** Microstates (dots) colored by their computed free energies from the MEMM analysis. Inset denotes ΔG scale in kcal/mol and is set relative to polymerization state.

Prior to estimating kinetic rates from our simulation data, we carried out time-lagged independent component analysis^33^ on the unbiased MD trajectories to identify the slowly varying degrees of freedom associated with the pol-to-exo conformational transition. Select atomic distances between the primer strand and the α/ε subunits (see Methods) were computed along the unbiased MD trajectories. Time-lagged independent components (ICs) were obtained from this distance data and all trajectory frames were projected onto the first two ICs (Fig. 3a). Under-sampled regions in the space defined by the two ICs indicated the presence of significant energy barriers in the pol-to-exo mode transition. Umbrella sampling was then selectively applied only to these barrier regions of the free energy landscape (Supplementary Fig. 4).

**Fig. 4:**
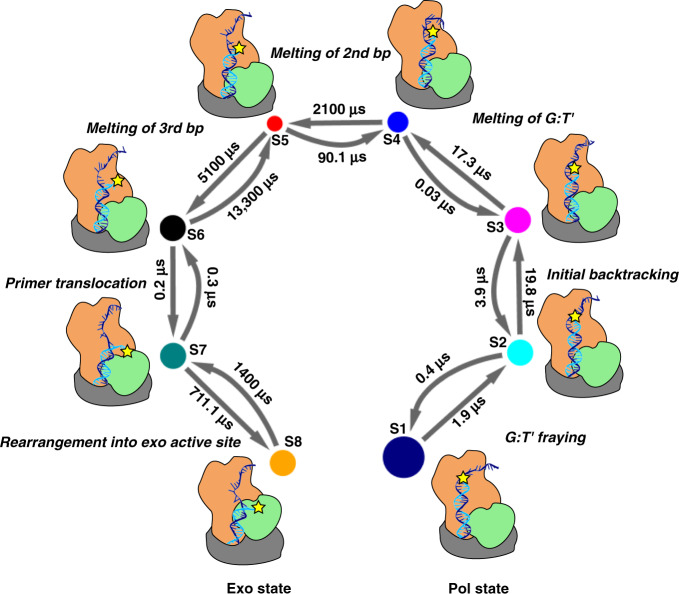
Complete kinetic model for the pol-to-exo mode transition connecting all on-path intermediates identified by the MEMM analysis. Macrostates S1–S8 are denoted by circles. Larger circles correspond to more populated macrostates. Transition between states are indicated with arrows and computed timescales for transitioning in and out of each macrostate are shown above the arrows. Each microstate is also represented by a cartoon, indicating the position and the extent of unpairing of the DNA primer end. The position of the mismatch nucleotide on the primer strand is indicated by a yellow star.

To combine the biased and unbiased MD data, we employed the transition-based reweighting analysis method (TRAM) in the PyEMMA package^29^, a recently introduced statistically optimal approach to estimate multi-ensemble Markov models (MEMM)^29,30^ with full thermodynamic and kinetic information at all ensembles. The approach combines the benefits of Markov state models^34–37^ – kinetics-based clustering of high-dimensional data and modeling of complex many-state systems – with the strength of biased MD to accelerate rare event sampling. The method has been shown to yield reliable microstate free energies and accurate kinetic rates on timescales of milliseconds to seconds, directly comparable to experiment^30^. We constructed an MEMM, which partitioned the conformational ensemble into eight kinetically distinct macrostates (denoted S1–S8, Fig. 3b and Supplementary Movie 2). We then computed probability fluxes and estimated transition timescales in and out of each macrostate. The end result was a complete kinetic model for pol-to-exo conformational switching (Fig. 4). Notably, we found that primer translocation to the exonuclease site occurs on an overall timescale of ten milliseconds, exceeding the timescale of nucleotide incorporation by an order of magnitude. This is the first estimate of the rate of primer strand switching from the polymerase to the exonuclease active site in Pol III. Thus, Pol III core achieves a delicate balance: the rate of conformational switching is slow enough not to interfere with normal nucleotide incorporation, and yet minor stalling upon mismatch encounter causes efficient transfer and removal of the incorrect nucleotide by the Pol III ε subunit. The effective free energy landscape along the two ICs (Fig. 3a) indicates a stepwise pol-to-exo mode transition with clearly resolved DNA melting and primer end translocation events. The process starts from state S1 (polymerization mode), proceeding through two early intermediates S2 and S3, in which the terminal G-T base pair is unraveled. While in state S2 the frayed primer end is still proximal to the polymerization site, in state S3 the mispaired T base has rotated away by 6 Å, effectively preventing DNA synthesis. Additional DNA translocation along the DNA axis by ~7 Å in S4 leads to an intermediate with completely open G-T base pair and partially disrupted hydrogen bonding for the second base pair from the primer end. DNA backtracking and rotation are facilitated by a patch of positively charged residues from the extended fingers domain (K839, R876, R877, and K881) that make contacts with the downstream DNA duplex. The highest barriers in the free energy landscape correspond to unpairing of the second and third nucleotide from the primer end (S4–S5 and S5–S6 transitions). The respective saddle point regions are 10.9 and 15.5 kcal/mol higher than the initial state S1 (Fig. 3c), resulting in the slowest computed timescales of 2100 μs and 5100 μs. Starting in S4, residues from the Pol III thumb domain insert between the template and the primer end serving as a wedge to separate the two strands. In states S5 and S6, a positively charged patch on the surface of the thumb domain (K439, R443, R447, and K461) binds and provides electrostatic stabilization for the transitioning DNA primer overhang. We also note the slow timescale for the back transition from state S6 to S5, which likely prevents backsliding of the system towards the pol state. The final stages of primer translocation (S6–S7 and S7–S8 transitions), involve tilting of the Pol III thumb domain away from the dsDNA and the π-stacking of a tyrosine (Y453) onto the last base pair of the DNA duplex. Together, these conformational shifts induce strain in the downstream DNA duplex and further increase the separation of the primer and template strands. In state S7, the terminal thymine base contacts a hydrophobic residue cluster from the ε subunit (M18, V65, and F102). Between states S7 and S8, we observe closing of the gap between the α and ε subunits, allowing the primer end to insert into the exonuclease active site in a catalytically competent orientation. The timescale for this transition is comparatively slow (711.1 µs and a free energy barrier of 8.2 kcal/mol), suggesting that primer insertion is gated by the motion of ε subunit within the Pol III core. Indeed, in previous studies the ε subunit was found to be relatively mobile during pol-to-exo switching due to the weak interactions of ε with the β clamp^38,39^.

When the primer strand is bound to the exonuclease active site, the large majority of the contacts are to the terminal nucleotide, with only two hydrogen bonds between the penultimate nucleotide and the protein (Supplementary Fig. 5)^40^. Therefore, once the bond between the terminal and penultimate nucleotide is cleaved, there are few interactions that keep the primer strand within the exonuclease. With the mispaired nucleotide removed and only two melted nucleotides remaining, the return to the polymerase active site will be swift, enabling DNA synthesis to resume without delay.

### Critical residues in the pol-to-exo conformational transition

**Fig. 5:**
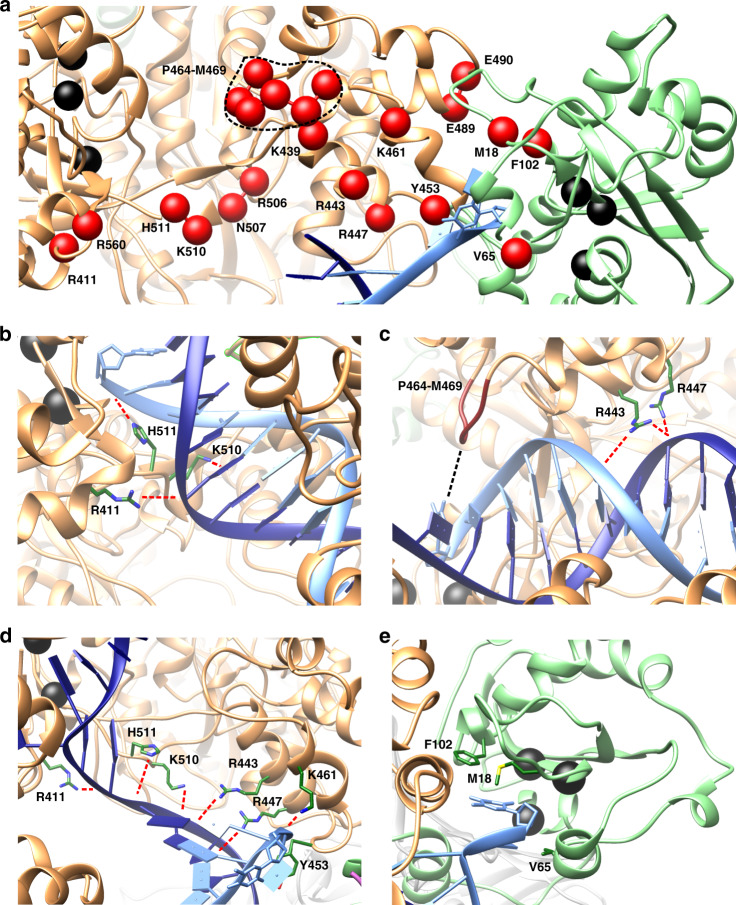
Specific interactions along the optimal path accommodate the transitioning primer end to ensure facile pol-to-exo switching. **a** Key residues (critical nodes) for pol-to-exo mode switching determined from dynamic network, conservation, and persistent contacts analyses and mapped onto the Pol III structure. Critical nodes are shown as spheres, labeled and colored in red. Polymerase and exonuclease active site residues are shown as spheres and colored in black. **b**–**d** Palm and thumb domain residues of the α subunit forming contacts important for polymerization (**b**, **c**) and for transitioning the primer end (**d**). Residue sidechains are shown in stick representation and labeled and colored by atom type (C is green, N is blue, and S is yellow). Salt-bridge and polar interactions to the DNA are shown as dashed red lines. Hydrophobic interactions are shown as a dashed black line. **e** Stabilization of the incoming mismatched nucleotide by the hydrophobic cluster of the ε subunit. Residues from the ε subunit hydrophobic patch are shown as sticks, labeled and colored in green.

The MEMM results allowed us to analyze each kinetically distinct macrostate and dissect the precise interactions, dynamic rearrangements and residue networks underlying the switching mechanism. Knowledge of the detailed mechanism served as a basis for successful validation of our computational models. Specifically, we employed dynamic network analysis to partition the holoenzyme complex into dynamic communities (tightly connected clusters of residues that move together as modules), mapping protein and nucleic acid residues onto graphs wherein each residue is a node and contacting nodes are connected by edges. All edges are weighted by dynamic correlation. Using these graphs, we computed suboptimal paths^41,42^ connecting the polymerization and exonuclease active sites for states S1–S8 (Supplementary Fig. 6). Suboptimal paths are a set of paths with length shorter than a specified limit above the optimal path. Suboptimal paths reflect residue correlations in molecular dynamics and, thereby, offer a way to quantify allosteric communication. Furthermore, nodes traversed by the largest number of suboptimal paths frequently correspond to critical residues for allosteric communication and regulation. Critical residues in the Pol III core identified by the above analysis were also tested for amino acid conservation and persistent contacts between DNA and the α or ε subunits. Combined residue scores were obtained from the individual suboptimal path score, conservation score and contact persistence score. Highest scoring residues were then mapped onto the Pol III holoenzyme structure (Fig. 5a and Methods).

Critical residues were found in the palm and thumb domains, for which we posit multiple roles in pol-to-exo conformational switching (Fig. 5b–d). In pol mode, residues R443, R447, and K510 form contacts to the DNA minor groove, while also stabilizing the separated template and primer strands during the latter stages of the transition. Pol III thumb domain residues Y453 and K461 stabilize the separated primer. The importance of Y453 was noted in previous experimental studies^7,43^. We also noted a loop in the thumb domain (P464-M469) that protrudes into the DNA major groove in polymerization mode, while directly binding the template strand in exonuclease mode. We posit that the P464-M469 loop restricts the movement of the DNA duplex during replication while during the pol-to-exo transition it serves to anchor the template strand, ensuring strand separation prior to exonuclease excision. Palm domain residues R411, H511, and R560 may serve similar roles, interacting with the DNA minor groove and the template strand. We also identified a hydrophobic cluster at the opening of the ε active site (M18, V65, and F102; Fig. 5e) that transiently stabilizes the primer end prior to insertion into the exonuclease site – a process which is dynamically gated by the motion of the ε subunit.

### Biochemical analysis of Pol III core mutants confirms an optimal transition path

**Fig. 6:**
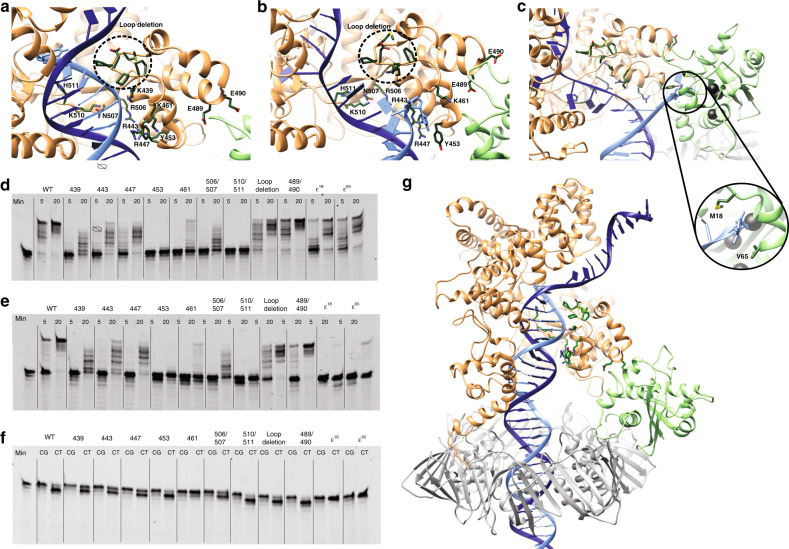
Residues experimentally determined to be critical for transfer of DNA primer strand from polymerase to exonuclease active site. **a**–**c** Close up of the transition path between polymerase and exonuclease active site in (**a**) polymerase mode, (**b**) intermediate mode, and (**c**) exonuclease mode. Polymerase is colored in orange, exonuclease in green, template DNA strand in dark blue, and primer strand in light blue. Mutated residues are shown in dark green sticks. **d** Denaturing gel analysis of polymerase activity of wild type and mutant proteins on matched DNA. Mutants showing W-T activity are highlighted in green, mutations that are moderately affected in orange, and mutations that render the protein inactive in red. **e** Similar analysis using a DNA substrate with a terminal C–T mismatch. **f** Exonuclease activity on matched (C–G) and mismatched (C–T) DNA measured in the same DNA substrates as in panels **d** and **e** in the absence of nucleotides. **g** Overview of Pol III core complex in polymerase mode. The mutated residues are highlighted in dark green and the β-clamp in gray. The experiments in panels **d**, **e** have been reproduced more than three times, the experiment in panel **f** have been reproduced twice.

To validate the defined path between the polymerase and exonuclease active site, we created eleven different mutations located in the vicinity of the polymerase and exonuclease active sites and along the path between the two sites. Seven of the mutations are located near the DNA in the palm or thumb domain (α^439^, α^443^, α^447^, α^453^, α^461^, α^506/507^, α^510/511^; Fig. 6a). One of the mutations is located distal from the polymerase active site at the interface of the thumb and exonuclease (α^489/490^). Two are located in the exonuclease at the entrance of the active site (ε^18^, ε^65^; Fig. 6b) A third exonuclease mutant ε^102^ was not soluble and, therefore, was excluded from the experiments. Finally, we also deleted a loop in the thumb domain (residues 464–469: α^loop^) that protrudes into the DNA major groove, seemingly pushing it down into the polymerase active site, yet having no direct contact with the DNA^8^.

All mutants were assembled into the trimeric polymerase-exonuclease-clamp complex and purified by gel filtration in 20 mM HEPES pH 7.5, 100 mM NaCl, and 2 mM DTT. To ensure a stable complex, an improved clamp-binder variant of the polymerase was used. This variant shows a >100-fold more stable complex than wild-type while retaining normal polymerase activity^8^.

Next, we analyzed the polymerase and exonuclease activity of all the mutant polymerase-exonuclease-clamp complexes on different DNA substrates and conditions (Fig. 6). On a matched DNA substrate (containing a C:G base pair at the terminal position; Fig. 6d) the two ε mutants and two α mutants located further away from the pol active site show no change in their activity compared with the wild type (ε^18^ and ε^65^, α^loop^, α^489/490^). The remaining α mutants show varying degrees of reduction in polymerase activity (α^439^, α^443^, α^447^, α^461^, α^506/507^) whereas two mutants are completely inhibited (α^453^, α^510/511^). None of the α mutations are part of the catalytic triad (composed of the three aspartates 401, 403, and 555^20^) but instead contact the DNA substrate backbone. Their reduced activity highlights the complexity of the polymerase active site and the necessity for accurate positioning of the DNA substrate for optimal polymerase activity.

Next, we tested the polymerase activity on a mismatched DNA substrate (containing a C:T mismatch at the terminal base pair), which requires the removal of the mismatched base before polymerase activity can proceed (Fig. 6e). The wild type and α mutants show no discernable difference in polymerase activity between the matched and mismatched substrate. In contrast, the exonuclease mutants ε^18^ and ε^65^ show an almost complete inhibition of activity on the mismatched DNA substrate. The ε mutants do not show significant difference in exonuclease activity when tested in isolation on a ssDNA (see Supplementary Fig. 7), indicating that the reduction of activity is unique to the pol-exo-clamp complex, and the required transition of the primer strand from pol to exo site.

As the reduced polymerase activity of the majority of α mutants is masking the exonuclease activity in the DNA extension assay, we isolated the exonuclease activity from the polymerase activity by omitting dNTPs from the reaction conditions and followed DNA excision (Fig. 6f). On a matched (C:G) substrate, no exonuclease activity was observed, indicating that the DNA is prevented from reaching the exonuclease site. In contrast, on a mismatched substrate (C:T) the wild type and several of the α mutants (α^453^, α^510/511^, α^489/490^, and α^loop^) show robust removal of the first nucleotide. In all, only the first nucleotide is removed while the isolated exonuclease on ssDNA show processive exonuclease activity, further supporting the observation that within the pol-exo-clamp complex the polymerase protects matched DNA from the exonuclease. The remaining α mutants that are located in the path between the pol and exo active site (α^439^, α^443^, α^447^, and α^506/507^) show decreased exonuclease activity on a mismatched DNA substrate, while α^461^ and the two ε mutants show a complete inhibition of exonuclease activity. As both wild-type ε and the exonuclease mutants ε^18^ and ε^65^ show near wild-type activity on ssDNA when measured in isolation (Supplementary Fig. 7), the reduced exonuclease activity of the pol-exo-clamp complexes indicates a defect in the transitioning of the primer strand from the polymerase to exonuclease active site. This is consistent with our MD analysis that predicted an essential role of these residues in the transfer of the primer strand between the two active sites.

## Discussion

Replicative DNA polymerases achieve their remarkable fidelity by striking a delicate balance between DNA synthesis and excision of mis-incorporated nucleotides from the growing primer strand. To efficiently switch between DNA synthesis and excision, these versatile enzymes confine each activity into distinct active sites^44^. To ensure facile transfer of the DNA primer between these spatially separated sites, the entire DNA polymerase holoenzyme reorganizes along a well-defined conformational path. In this contribution, we combine state-of-the-art computational methods with closely coupled biochemical analyses to determine the optimal free energy path connecting the polymerization and exonuclease states of bacterial Pol III holoenzyme. We also use new data mining and classification strategies to discover kinetic intermediates, compute transition timescales and define molecular mechanisms based on analysis of the simulated Pol III conformational ensemble. Importantly, our results delineate a complete pol-to-exo mode switching mechanism addressing structural intermediates, protein dynamics, free energies, and kinetics of the Pol III holoenzyme. All aspects of the mechanism emerge from our data analysis without a priori assumptions.

Our predictive mechanism involves stepwise melting of the first three nucleotides from the DNA primer end. Fraying of the mismatched terminal base pair is facile and occurs on a microsecond timescale at the earliest stages of the transition. The departure of the terminal thymine base from the polymerase active site results in a stalled polymerase state. Next, the Pol III holoenzyme exploits the natural motion of the DNA inside the Pol III/β-clamp central cavity to backslide and rotate, completely releasing the primer-template junction from the polymerase active site. The motion is guided by residues from the Pol III thumb and fingers domains. Base unpairing at the second and third position from the primer end is progressively more energetically costly. Thus, the second and third unpairing events result in the highest barriers along the path and bring the overall timescale for the pol-to-exo transition into the millisecond range. High-energy intermediates on the landscape (plateau regions corresponding to S4 and S5) are stabilized by interactions with thumb domain residues, preventing backsliding toward pol mode. Notably, once base unpairing is complete the ssDNA primer undergoes fast sliding along the surface of the thumb domain, facilitated by contacts with strategically positioned residues. Consistent with their proposed mechanistic roles, mutations of these residues (e.g. α^453^, α^461^) slow down but do not abolish exonuclease activity. The fast rate of primer translocation compared to DNA melting has been noted in previous experimental studies and appears to be conserved across the A, B, and C-family polymerases^39,45–48^. The DNA primer’s initial binding to the ε subunit is also a crucial step in ensuring efficient transfer. We identify a hydrophobic residue cluster that serves to stabilize the primer end prior to exonuclease site insertion. Importantly, we show that site mutations disrupting the cluster (ε^18^, ε^65^) affect exonuclease activity. Finally, we note the conformational shift and increased mobility of the ε subunit are essential features of our proposed mechanism. Insertion of the primer terminus into the exonuclease active site is gated by the motion of the ε subunit highlighting the important role of protein dynamics in the pol-to-exo mode transition.

The principles of the strand transfer are likely to be conserved in the closely related PolC and DnaE polymerases. In addition, the structurally distinct A-family polymerases (Pol I, T7) and B-family polymerases (Pol II, human Pol δ and ε) are likely to employ a similar approach for the strand transfer as well. Like Pol III, the A- and B-family polymerases also have their exonuclease located ~60 Å away from the polymerase active site and show a three-nucleotide fraying of the primer strand when binding to the exonuclease active site^32,49^. Hence, for these polymerases too there will be a requirement for an optimal path between pol and exo site.

In summary, our results shed light on the sophisticated strategies that allow replicative polymerases to achieve their extraordinary precision. Combining advanced computational modeling with insightful validation experiments, our study contributes to integrated understanding of high-fidelity DNA polymerases as dynamic assemblies engaged in safeguarding genome integrity.

## Methods

### Model building and equilibration

Pol III holoenzyme models (Pol III/β-clamp/primer-template DNA) were constructed in polymerization and editing modes from the available cryo-EM structures (PDB and EMDB accession codes 5M1S and 5FKV; EMD-4141 and EMD-3198)^7,8^. The last base of the primer strand was converted to a thymine to produce a G:T′ mismatch (′ indicates primer strand). The DNA construct in the original cryo-EM structure had a four-nucleotide overhang (5′-TCAG) shown to be important for exonuclease activity^7^. In our models we extended the template strand by an additional four nucleotides to 5′-TCAGTCAG. This ensured that the overhang spanned the surface of the polymerase and also extended into solvent. Missing residues in the ε flexible linker connecting the C-terminus with the catalytic domain were built using the ModLoop utility in the Modeller package^50^. All simulations were carried out with the Amber *ff14SB* force field parameters^51^ using the NAMD molecular dynamics code^52^. Electrostatics were calculated using the smooth particle mesh Ewald method and non-bonded interactions were evaluated with a 10-Å cutoff and 8.5-Å switching distance. Models were solvated with TIP3P water molecules from an equilibrated solvent box, ensuring 10 Å padding from the protein or nucleic acid atoms to the edge of the simulation box. Na^+^ and Cl^−^ counterions were added to neutralize the overall charge of the Pol III holoenzyme complex and bring the ionic concentration to 150 mM. Both simulation systems were subjected to energy minimization for 5000 steps using the conjugate-gradient and line search algorithm and equilibrated for 5 ns with molecular dynamics flexible fitting (MDFF)^53^ to ensure conformance to the respective cryo-EM densities. In the first stage of MDFF models were gradually heated to 300 K in the NVT ensemble while enforcing positional restraints on all heavy atoms using a force constant of 5.0 kcal mol^−1^ Å^−2^. Positional restraints were then incrementally decreased to 0.0 kcal mol^−1^ Å^−2^ in the NPT ensemble (1 atm and 300 K). A scaling factor of *ξ* = 0.1 was employed during all stages of MDFF. By simultaneously decreasing positional restraints and enforcing weak MDFF grid forces, both systems were allowed to gradually relax into their respective EM densities. The MDFF simulations employed a 1-fs timestep.

### Path optimization protocol

A short 10-ns targeted molecular dynamics (TMD) run was used to connect the equilibrated end states. The exo-mode conformation was selected as the TMD target and the pol-mode conformation was driven to the target with a force constant of 1000 kcal mol^−1^ Å^−2^. From the TMD trajectory we selected 32 evenly spaced snapshots (replicas) at 312-ps intervals that served to initiate our path optimization protocol, employing the partial nudged elastic band method (PNEB)^25,26^. The optimization protocol was carried out in several stages. First, replicas were heated to 300 K for 1.5 ns while employing a 20-kcal mol^−1^ Å^−2^ PNEB force constant. This was followed by a 3-ns run at 300 K using a 10-kcal mol^−1^ Å^−2^ force constant. For the subsequent 1.5 ns the chain-of-replicas was cooled to 0 K using a force constant of 20 kcal mol^−1^ Å^−2^. This annealing cycle was repeated two more times to allow the replicas to gradually spread along the path and relax into local minima. PNEB path convergence was monitored by computing the change in RMSD values for all the replicas after each round of annealing. Changes <0.3 Å RMSD from the previous conformations for all replicas in the band were considered sufficiently converged. The initial and final replicas were excluded from optimization to ensure conformance to the observed pol and exo conformations from cryo-EM. All protein and DNA heavy atoms were included in the PNEB calculation. The CUDA PMEMD module of the Amber molecular dynamics package was used for these simulations^54–56^.

### Unbiased and biased sampling along the minimal energy path

To sample extensively the conformational states along the optimal path, we initiated unbiased molecular dynamics simulations from each of 32 optimized replicas. Replicas were heated to 300 K for 500 ps in the NVT ensemble while imposing 5 kcal mol^−1^ Å^−2^ positional restraints on all heavy atoms. The restraints were scaled down to 0 kcal mol^−1^ Å^−2^ in a 5-ns NPT run. Each replica was then simulated for 200-ns using free unbiased MD, resulting in 6.4 μs of aggregate sampling along the PNEB path. All production runs were executed in the NPT ensemble (1 atm and 300 K) with a 2-fs timestep using the CUDA PMEMD module of the Amber code. VMD and UCSF Chimera packages were used for analysis and visualization^57,58^.

To improve sampling of regions in conformational space inaccessible to free unbiased MD we used umbrella sampling (US). These regions corresponded to barrier or high-energy plateau regions of the free energy landscape. We used a distance-based reaction coordinate (RC) for US biasing. Specifically, we selected the center-of-mass distance between the three nucleotides from the primer end and the exonuclease active site residues (D12, E14, and D167). The RC was subdivided into 12 overlapping windows with 1.0-Å spacing. Each window was simulated for 25 ns employing a force constant of 15 kcal mol^−1^ Å^−2^. Umbrella sampling trajectories were then projected onto the first two eigenvectors obtained from time-lagged independent component analysis (TICA; refer to next section for details)^33,59^. All umbrella sampling simulations were performed in the NPT ensemble (1 atm and 300 K) using the NFE module of AMBER. Configurations from the center of each umbrella window were then used as seeds for short (50-ns) unbiased MD simulations (Supplementary Fig. 4). This was done to ensure that the barrier regions contained both biased and unbiased sampling, a requirement for TRAM.

### Time-lagged independent component analysis

To identify slowly varying degrees of freedom associated with the pol-to-exo conformational transition, we carried out dimensionality reduction on the trajectory data using time-lagged independent component analysis (TICA)^33,59^. Atomic distances between the first 10 base pairs of dsDNA and protein residues on the α/ε subunit were selected as collective coordinates for TICA. Residues from α/ε were selected by computing all protein contacts within 5 Å of the first 10 base pairs of dsDNA across all configurations in the MEP. Phosphorous atoms on the backbone of each nucleotide and Cα atoms from each α/ε amino acid were used as a reference to compute the Euclidean distance between residues. Additionally, we included distances between N1 and N3 atoms on the first three base pairs that split and form the separated primer strand. In total, 456 unique distances were selected for dimensionality reduction with TICA. A lag time of *τ* = 500 ps was used to compute the time-lagged covariance matrix. This matrix was then diagonalized to produce the respective eigenvectors and eigenvalues. MD trajectories were then projected onto the first two eigenvectors to yield the time-lagged independent components (ICs).

### Multi-ensemble Markov Model estimation

Combining unbiased and biased simulation data allowed us to sample and achieve uninterrupted coverage of the transition path space defined by the first two ICs. K-means clustering was then employed in projected IC space producing 1000 microstate clusters. We then employed the transition-based reweighting analysis method (TRAM) to analyze our biased and unbiased simulation data producing correct free energy weighting of our microstates. A lag time of 500 ps was selected for the TRAM estimator based on the relaxation time of the estimated implied timescales (Supplementary Fig. 8). Kinetically similar microstates were then agglomerated into the S1–S8 macrostate clusters using the PCCA + algorithm^60^. Transition timescales were computed between macrostates using transition path theory^61^ resulting in a kinetic model for primer translocation through the Pol III holoenzyme complex. Reported timescales represent mean first passage times (i.e. average time it takes a trajectory to leave one state and enter another).

### Bootstrapping

In order to compute error bars associated with the transition timescales and microstate free energies a bootstrap was performed. Prior to bootstrapping, multiple independent simulations were initiated from all 32 configurations along the MEP and from the center of each umbrella window. For each bootstrap sample one TRAM estimation was performed. Samples were generated by combining a simple bootstrap with a stationary bootstrap^62^. Under this paradigm, whole trajectories from the unbiased simulation data are drawn with replacement, while trajectory blocks of random length from the biased simulation data are drawn according to the stationary bootstrap algorithm. The minimum block length was selected to be the mean statistical inefficiency of the discretized trajectories in the umbrella sampling data set (5 ns). Error bars for the transition timescales and the microstate free energies in the barrier regions are presented in Supplementary Tables 1 and 2.

### Critical residues in the Pol III holoenzyme dynamic network

Dynamic network analysis was used to map protein and nucleic acid residues onto graphs wherein each residue is a node and contacting nodes are connected by edges (see Supplementary Fig. 6). All edges are weighted by dynamic correlation. Dynamic networks were constructed using NetworkView^63^. Using these graphs, we computed suboptimal paths^41,42^ connecting the polymerization and exonuclease active sites for states S1–S8. Sampling of 50,000 frames from each macrostate were selected for this structural analysis. Suboptimal paths are a set of paths with length shorter than a specified limit above the optimal path. Suboptimal paths reflect residue correlations in molecular dynamics and, thereby, offer a way to quantify allosteric communication. Nodes traversed by the largest number of suboptimal paths frequently correspond to functionally important residues in the biological complex. In all, 150,000 paths connecting α^D403^ and ε^D167^ were calculated for each macrostate S1–S8 using the Floyd-Warshall algorithm on the weighted network graphs and a distance cutoff of 30 Å. Weights were defined as −ln|*c*_*ij*_|; *c*_*ij*_ are correlation coefficients between residues. We then normalized the distribution of critical residues across all macrostate suboptimal pathways. In addition to occupying privileged positions in the dynamic network we also require candidate residues to be conserved and to be in persistent contacts with the first 10 dsDNA pairs for at least part of the pol-to-exo conformational transition. It was recently suggested that the *E. coli* polymerase is a phylogenetic outlier due to its ε-subunit^13^. Moreover, the *E. coli*-like exonuclease appears to exist explicitly in alpha-, beta-, and gamma-proteobacteria leading us to only include these three groups in our conservation analysis. Amino acid conservation scores were determined using the EVcouplings server^64^ and mapped to the structure of Pol III (Supplementary Fig. 9). Finally, we determined contacts between Pol III and the first 10 dsDNA base pairs for all macrostates S1–S8. Protein residues were considered in contact if they were within 5 Å of the first 10 base pairs of the dsDNA. Contact persistence was computed as the frequency of appearance of the contact in the MD trajectories within the distance cutoff. Persistence values for each contact were then summed across all eight macrostates. Scores were obtained for each residue by combining their suboptimal path score, conservation score, and contact persistence score. From the combined scores (Supplementary Table 3) we selected the top 16 top scoring residues as candidates for experimental testing and validation.

### Protein purification and complexes assembly

All chemicals were purchased from Sigma Aldrich or Fisher Scientific, DNA oligonucleotides from Sigma and chromatography columns from GE Healthcare. Site direct mutagenesis was used to create nine mutants of the DNA Polymerase III α subunit and two mutants of the exonuclease ε (Supplementary Table 4). All proteins were expressed in *E. coli* BL21 (DE3). The α mutants were purified using a Histrap, Hitrap Q, and a HiLoad Superdex 200 (120 ml) column. The β clamp was purified with a Histrap and a Hitrap Q column. The two exonuclease mutants ε^18^ and ε^65^ where purified from inclusion bodies in 6 M Urea using a Histrap column. The protein was then refolded by overnight dialysis into 0 M Urea and subsequently loaded on a Hitrap Q column. A third exonuclease mutant ε^102^ did not refold into soluble protein as was excluded from the studies. To assemble the complexes α, β, and ε were mixed in a ratio 1:1.5:1.5, respectively, and loaded on a Superdex 200 Increase (2.4 ml) column. After 12% SDS PAGE gel analysis, fractions that contained the three proteins were pooled together. The individually created mutant complexes were further analyzed by SDS Page using 4–20% Mini-PROTEAN TGX Precast Protein Gels to confirm all complexes were at the same concentration. All proteins and complexes were flash frozen in liquid nitrogen and stored at −80 °C.

### DNA Primer extension assay

Polymerase and exonuclease activities were measured using a 26 base pair dsDNA substrate with a 11-nucleotide single-stranded overhang. (template strand: 5′ -GCTAGCTTACACGAGTCCTTCGTCCTAGTACTACTCC-3′; matched primer strand: 5′-6-FAM GGAGTAGTACTAGGACGAAGGACTCG-3′; and mismatched primer strand: 5′-6-FAM GGAGTAGTACTAGGACGAAGGACTCT-3′). All the reactions were performed at room temperature in 20 mM Hepes pH 7.5, 2 mM DTT, 5 mM MgCl_2_, 50 mM NaCl, and 0.5 mg/ml BSA. For the experiments with nucleotides 100 μM dNTPs (each) were added to the buffer. Reactions were started by addition of 40 nM of protein complex to 50 nM of DNA (final concentrations). Reactions with dNTPs were stopped at 5 and 20 min, while reactions without dNTPs were stopped at 5 min. All the reactions were performed with matched (CG) and mismatched DNA (CT). Reactions were then run on a denaturing 20% Acrylamide (19:1) gel in 1xTBE with 6 M Urea for 1 h and 20 min at 30 W. Afterwards the gel was imaged on Typhoon using Alexa Fluor 488 filter.

### Reporting summary

Further information on research design is available in the Nature Research Reporting Summary linked to this article.

## Supplementary information

Supplementary Information

Description of Additional Supplementary Files

Supplementary Movie 1

Supplementary Movie 2

Reporting summary

## Data Availability

Other data that support the findings of this study are available from the corresponding authors upon reasonable request.
