# Supplementary material for: Polymerization and editing modes of a high-fidelity DNA polymerase are linked by a well-defined path
Source: Nat Commun. 2020 Oct 23;11:5379. doi: 10.1038/s41467-020-19165-2 (PMC7584608; doi:10.1038/s41467-020-19165-2)
Supplement: Supplementary file 1 — Supplementary Information [file 41467_2020_19165_MOESM1_ESM.pdf]

Supplementary Information

Supplementary Figures

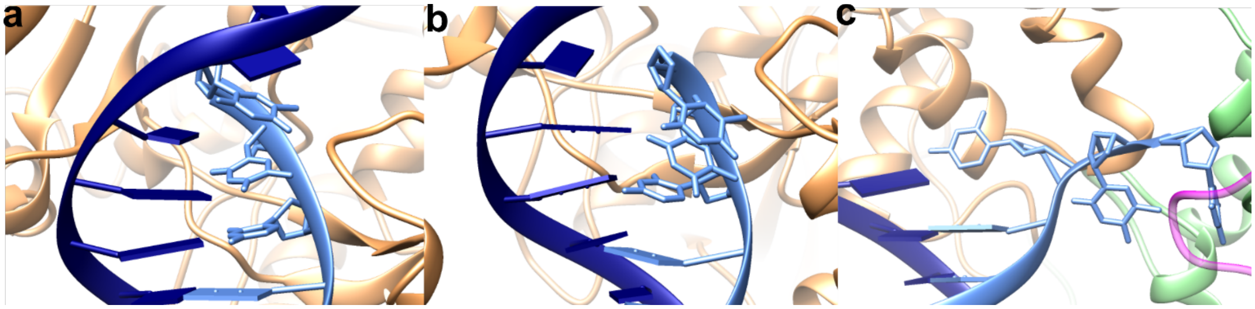

Supplementary Figure 1. Sequential unpairing of the first 3 base pairs during the pol-to-exo conformational transition. a, Melting of mismatched G:T' at primer terminus. **b**, Unpairing of the second base pair from the primer end. **c**, Melting of the third base from the primer end. The α subunit is colored in orange and the ε subunit is colored in green. The primer strand is colored in light blue; the template strand is colored in dark blue.

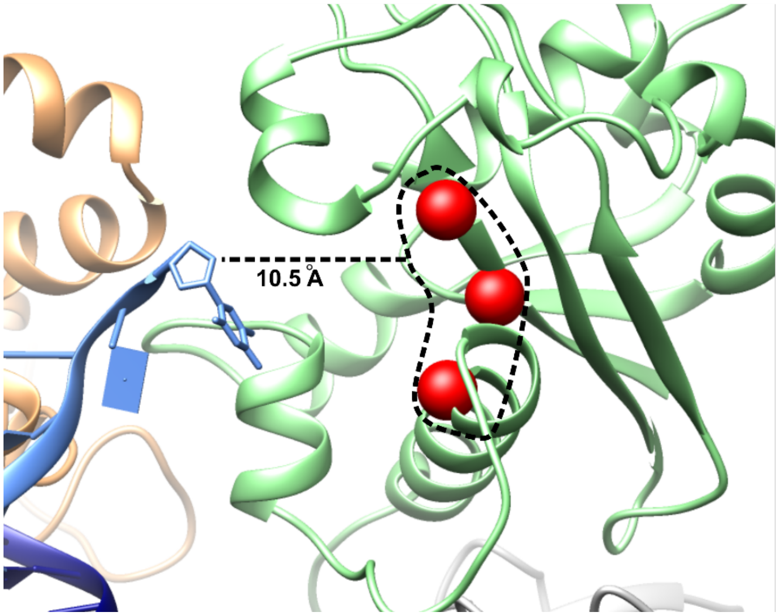

Supplementary Figure 2. DNA melting alone is insufficient for the primer end to reach the exonuclease active site. dsDNA from the exo state superimposed on Pol III holoenzyme conformation in the pol state. Distance measured from 3'-OH on mismatched nucleotide to the center of the exonuclease active site residues (red spheres) is shown by a dashed black line.

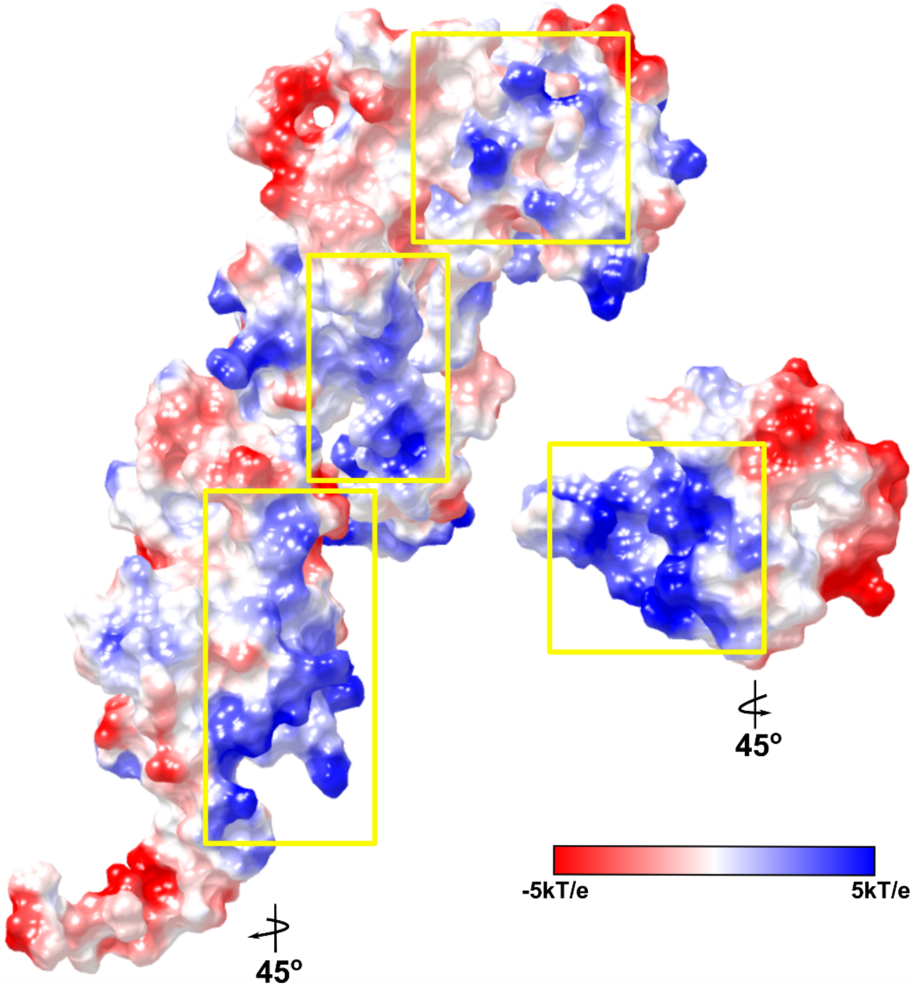

Supplementary Figure 3. Coulombic potential mapped onto the molecular surface of the Pol III α subunit. The positively charged surface of the palm, fingers (left) and thumb (right) domains provide electrostatic stabilization to accommodate the negatively charged DNA substrate. Regions of the α subunit that interact with the DNA template and primer strands are highlighted with yellow boxes. Domains have been rotated 45° away from each other. The PHP domain has been omitted for clarity.

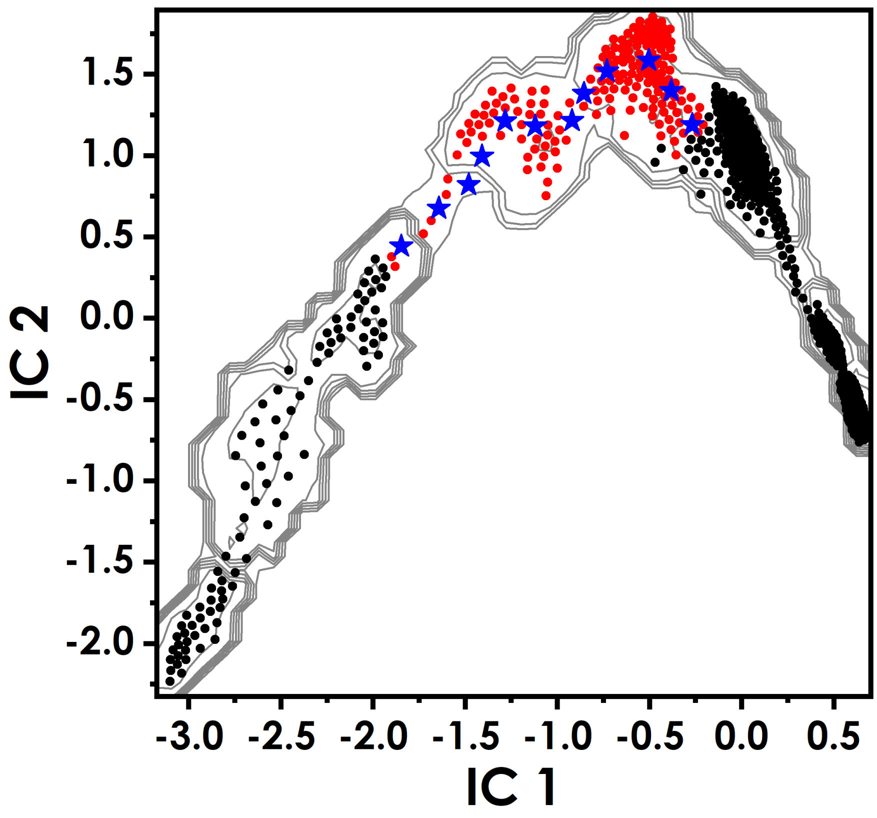

Supplementary Figure 4. Umbrella sampling simulations were used to overcome the energy barriers along the pol-to-exo conformational transition. 2D histogram of the MD simulation conformers projected on the independent components, IC 1 and IC 2, as determined from TICA analysis. Microstates determined by k-means clustering of the simulation trajectories are shown as dots. Microstates containing configurations from unbiased MD are shown as black dots. Microstates containing configurations from biased (umbrella sampling) and unbiased MD are shown as red dots. Configurations corresponding to the centers of the umbrella windows are denoted by a blue star.

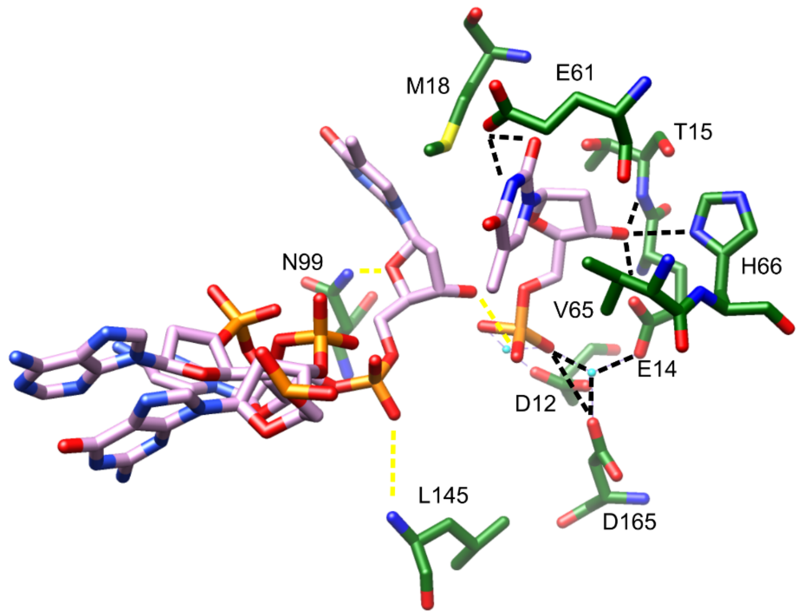

Supplementary Figure 5. **In the exonuclease state, the terminal nucleotide of the primer strand provides most of the contacts to the exonuclease active site.** Active site residues are labeled and color-coded according to atom type (C is dark green, O is red, N is blue, S is yellow, P is orange). The C atoms of the primer end are shown in violet to distinguish the primer from the protein residues. Important interactions to the primer are indicated with dashed lines.

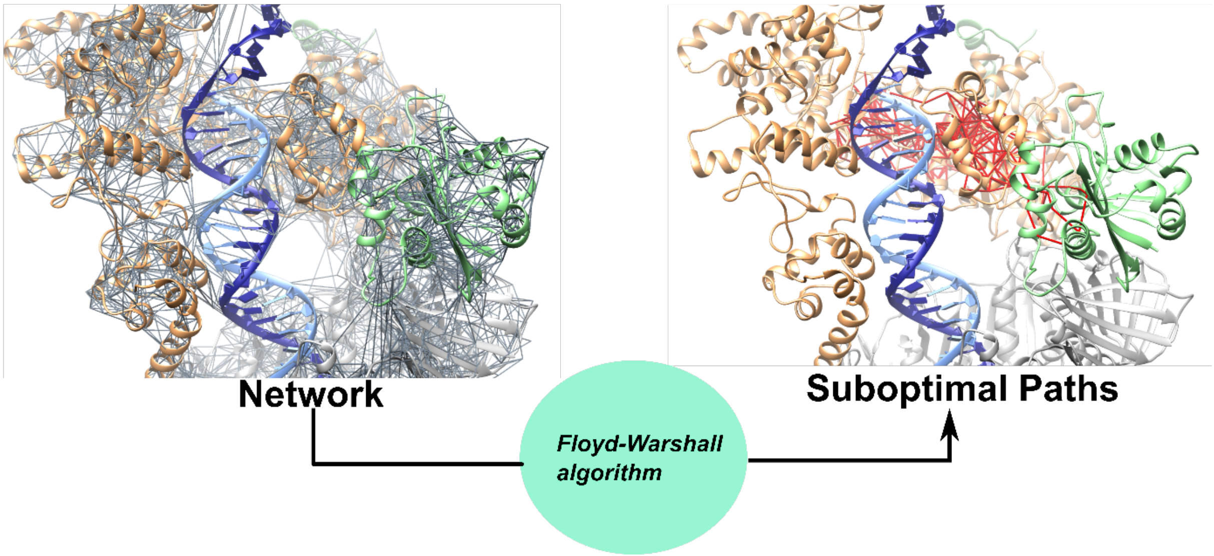

Supplementary Figure 6. Representation of the protein complex as a dynamic network and schematic indication of how suboptimal pathways are obtained from the dynamic network. Dynamic network (left) computed for the Pol III core-β complex using dynamic correlation as a measure of residue communication. Each Cα and P atom is a node in the network. Edges between nodes (gray edges in the left panel) are weighted by their dynamic correlation. All edges are shown with uniform width for clarity. From the dynamic network, the Floyd-Warshall algorithm is used to determine the suboptimal pathways (red edges in the right panel) between the polymerization and exonuclease active sites.

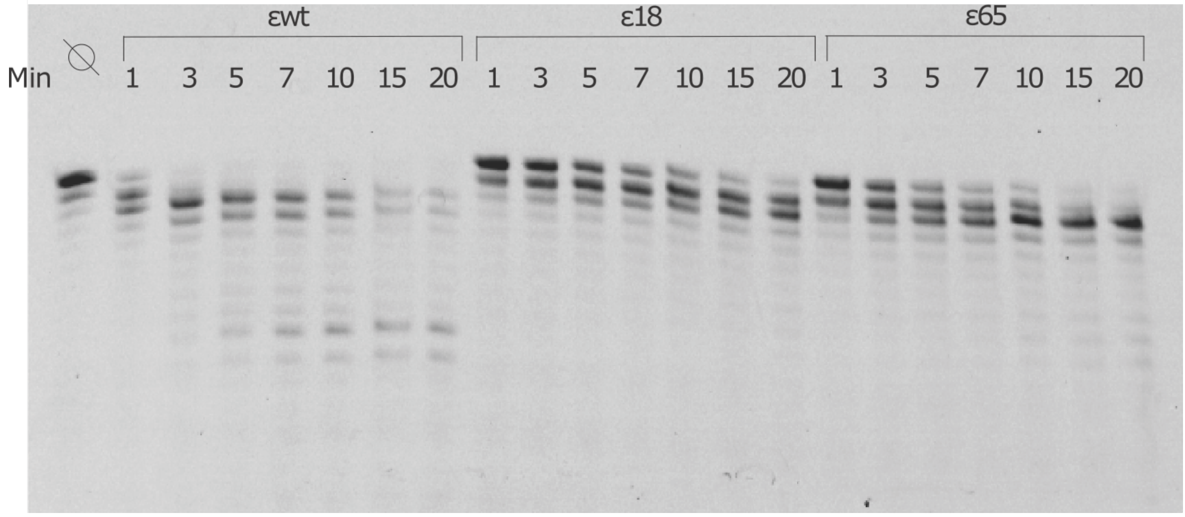

Supplementary Figure 7. Exonuclease activity of the isolated exonuclease on ssDNA. 3'-5' exonuclease activity of isolated wild type and exonuclease mutants (ε^{18} and ε^{65}) on a 26-nucleotide ssDNA. Both mutants show activity that is only moderately reduced compared to the wild type protein. The experiment was repeated two times and replication was successful.

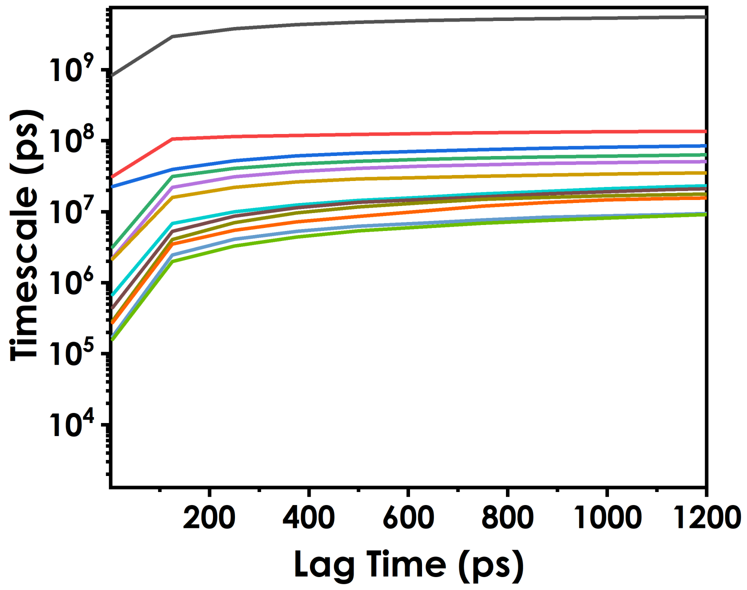

Supplementary Figure 8. Implied timescales as a function of lag time from analysis of biased and unbiased MD trajectories. Colors in the plot are arbitrary and aid in the visualization of the slowest timescales. Gaps separating the slowest implied timescales were used to estimate the number of kinetically distinct macrostates for MEMM construction. Only the first 10 timescales are shown for clarity.

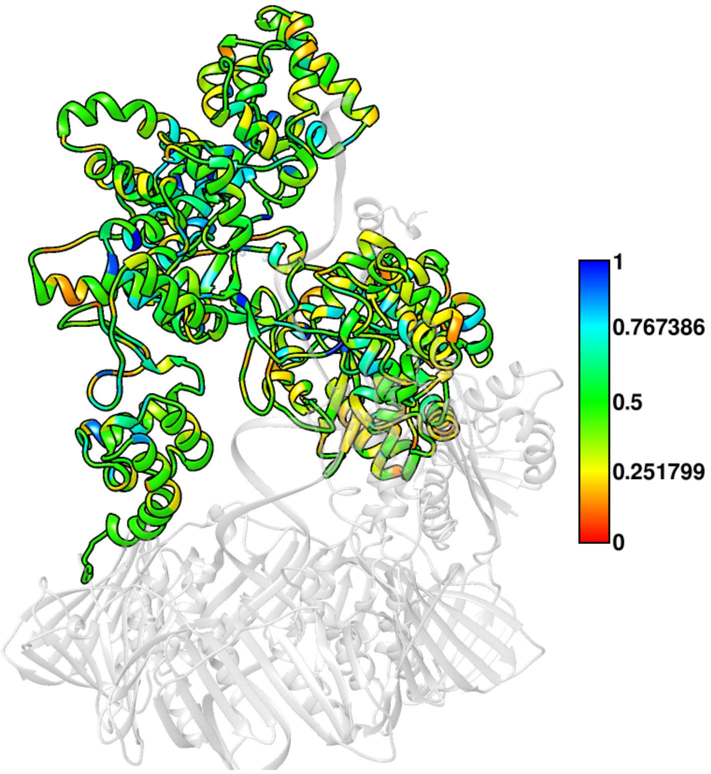

Supplementary Figure 9. Conservation map for the α subunit of Pol III. Conservation scores determined using the EVCouplings server are mapped onto the α subunit of the Pol III holoenzyme. Inset denotes normalized conservation scores.

Supplementary Tables

Supplementary Table 1. Associated errors of the transition timescales determined with TRAM.

Macrostate Transition	Timescale (μs)	StdErr +/- (μs)
S1 → S2	1.9	0.08
S1 ← S2	0.4	0.01
S2 → S3	19.8	0.03
S2 ← S3	3.9	0.02
S3 → S4	17.3	0.01
S3 ← S4	0.03	0.001
S4 → S5	2100	1.2
S4 ← S5	90.1	0.1
S5 → S6	5100	2.2
S5 ← S6	13300	12.8
S6 → S7	0.2	0.03
S6 ← S7	0.3	0.01
S7 → S8	711.1	0.8
S7 ← S8	1400	1.3

Supplementary Table 2. Associated errors of the microstate free energy estimates determined with TRAM.

Macrostates	ΔG (kcal/mol)	StdErr +/-
S4:S5	9.33	0.32
S4:S5	10.41	0.37
S4:S5	10.96	0.43
S5:S6	13.14	0.79
S5:S6	15.92	0.66
S5:S6	15.83	0.26
S5:S6	15.54	0.29
S5:S6	16.01	0.56
S5:S6	16.18	1.02

Supplementary Table 3. Critical residue scores determined from combining suboptimal paths, conservation and contact persistence data.

Residue	Subopt Distribution	Conservation Score	Summed Contact Persistence	Total
R411α	0	0.84	1.2	2.0
K439α	0.20	0.50	3.8	4.5
R443α	0.35	0.67	2.4	3.4
R447α	0.29	0.48	1.6	3.0
Y453α	0.35	0.45	2.8	3.7
K461α	0.54	0.35	1.8	2.7
E489α	0.90	0.38	0.0	1.3
E490α	0.68	0.45	0.0	1.1
R506α	0.10	0.94	1.3	2.3
N507α	0.17	0.42	1.1	1.7
K510α	0.15	0.32	5.8	6.3
H511α	0.05	0.56	4.1	4.7
R560α	0	0.80	4.0	4.8
M18ε	0.41	N/A	1.8	2.2
V65ε	0.37	N/A	1.7	2.1
F102ε	0.25	N/A	1.7	2.0

Supplementary Table 4 – DNA primers

Mutation	Primer 5'-3'
Lys439Ala	ACAATGGCGGCG**GCA**GCGGTGATCCG
Arg443Ala	AAAGCGGTGATC**GCC**GACGTAGGCCG
Arg447Ala	GCGACGTAGGC**GCC**GTGCTGGGGC
Lys461Ala	GATCGTATCTCG**GCA**CTGATCCCGCC
Tyr453Ala	CGATCGACAAAGCCG**GCC**GGATGCCCCAGCAC
Arg:Asn506:507Ala:Ala	CTGGAAGGGGTCACC**GCTGCC**GCCGGTAAGCAC
Lys:His510:511Ala:Ala	GTAACGCCGGT**GCGGCC**GCCGGTGGGGT
Glu:Glu489:490Ala:Ala	GAAGCGGAT**GCAGCA**GTTAAGGCGCT
464PPDPGM469; 464-GSG--466	Fw **GATCGGCAGCGG**GACGCTGGCGAAAGCGTTTGAAG<br>Rv **GTCCCGCTGCC**GATCAGTTTCGAGATACGATCGAC
Met18Ala	GAAACCACCGGT**GCG**AACCAGATTGGTGCG
Val65Ala	GGAAGCCTTTGGC**GCA**CATGGTATTGCCGATG
