# Supplementary material for: Polymerization and editing modes of a high-fidelity DNA polymerase are linked by a well-defined path
Source: Nat Commun. 2020 Oct 23;11:5379. doi: 10.1038/s41467-020-19165-2 (PMC7584608; doi:10.1038/s41467-020-19165-2)
Supplement: Supplementary file 2 — Description of Additional Supplementary Files [file 41467_2020_19165_MOESM2_ESM.pdf]

Description of Additional Supplementary Files

File Name: Supplementary Movie 1
Description: Minimum free energy path for conformational switching from polymerization to exonuclease mode for the Pol III holoenzyme complex. Individual subunits are colored as follows: a is orange; ε is light green; theta is magenta; primer DNA strand is light blue; template DNA strand is dark blue; mismatched T base at the primer end is red; pol and exo active site residues are shown as black spheres. The PHP domain, portions of the fingers domain and the θ subunit were omitted in the latter half of the video to ensure unobstructed view of the primer.

File Name: Supplementary Movie 2
Description: Kinetically distinct intermediates along the pol-to-exo mode transition identified from the multi-ensemble Markov model. Individual subunits are colored as follows: α is orange; ε is light green; θ is magenta; primer DNA strand is light blue; template DNA strand is dark blue; mismatched T base at the primer end is red; pol and exo active site residues are shown as black spheres. Amino acid residues from the α and ε subunits that contact the dsDNA during the transition are colored by atom type (C is dark green, O is red, N is blue, S is yellow) and shown in stick representation.
